# Is an HIV vaccine achievable? - Pros

**DOI:** 10.1186/1471-2334-14-S2-S14

**Published:** 2014-05-23

**Authors:** Marc Girard

**Affiliations:** 1 French National Academy of Medicine and Denis Diderot University, Paris, 75006, France

## 

The development of an HIV/ AIDS vaccine has been a long and difficult endeavor, which so far was met with a great many failures. New, recent advances may however change the deal. The RV144 clinical trial in Thailand showed that a canarypox-gp120 prime-boost vaccination regimen had a modest 31% efficacy at 42 months after vaccination, but protection was as high as 60% at 6 months after vaccination. A series of new trials is expected to take place in the near future to try to improve these numbers using a more potent vector than canarypox, gp140 rather than gp120, a water-in-oil adjuvant rather than aluminium salts, and increasing the number of booster immunizations. Protection in the RV144 trial correlated with the induction of antibodies (Ab) to the V2 region of gp120, not with neutralizing Abs. A major difficulty met in the development of an efficacious HIV vaccine has indeed been to design proper immunogens able to elicit broadly neutralizing Abs (bnAbs) that can neutralize a wide range of circulating virus strains. BnAbs are highly protective, as demonstrated by passive immunization experiments and by vectored immunoprophylaxis experiments using AAV as a vector in animal models such as macaques or humanized mice. Their efficacy in humans should be tested soon in upcoming clinical trials. On another hand, the report that monkeys immunized with a simian CMV-SIV vaccine can clear SIV infection following viral challenge has emphasized the importance of self-renewing, long-lasting memory T_EM_ cells that can differentiate into specific effector T cells and control provirus activation, eventually resulting in the clearance of infection. A HIV vaccine that could elicit both a bnAb response and the formation of specific T_EM_ cells would obviously be an ideal vaccine. It might even offer hopes for a cure of HIV infection when used therapeutically in HIV-infected persons. The development of such a vaccine is still long and difficult but may not to be an unrealistic goal.

